# Bilobar spreading of colorectal liver metastases does not significantly affect survival after R0 resection in the era of interdisciplinary multimodal treatment

**DOI:** 10.1007/s00384-012-1455-1

**Published:** 2012-03-21

**Authors:** K. Homayounfar, A. Bleckmann, L. C. Conradi, T. Sprenger, T. Beissbarth, T. Lorf, M. Niessner, C. O. Sahlmann, J. Meller, H. Becker, T. Liersch, B. M. Ghadimi

**Affiliations:** 1 Department of General and Visceral Surgery, University Medical Center, Georg August University Göttingen, Robert-Koch-Strasse 40, 37075 Göttingen, Germany; 2 Department of Hematology and Oncology, University Medical Center, Georg August University Göttingen, Göttingen, Germany; 3 Department of Medical Statistics, University Medical Center, Georg August University Göttingen, Göttingen, Germany; 4 Department of Nuclear Medicine, University Medical Center, Georg August University Göttingen, Göttingen, Germany

**Keywords:** Bilobar colorectal liver metastases, Liver resection, Multimodal treatment, Two-stage resection

## Abstract

**Purpose:**

Bilobar colorectal liver metastases (CRLM) are often considered incurable or associated with poor prognosis even after R0 resection. In this single-center study, we evaluate the impact of CRLM spreading on recurrence-free survival (RFS) and cancer-specific overall survival (CSS) after R0 resection of CRLM with respect to multimodal treatment strategies including perioperative chemotherapy and multistep resections.

**Methods:**

Between January 2001 and December 2010, R0 resection could be achieved in 70 patients with bilobar and 100 with unilobar CRLM. Extent of disease, perioperative chemotherapy, surgical procedures, adjuvant treatment, histopathological workup, RFS, and CSS were compared between both cohorts.

**Results:**

Forty-six (66 %) patients with bilobar and 26 (26 %) patients with unilobar CRLM received preoperative chemotherapy (*p* < 0.001). For bilobar CRLM, more extended and multistep resection including portal vein occlusion were performed (29 % versus 3 %; *p* < 0.001). Morbidity (39 % versus 28 %, *p* = 0.183) and mortality (1 % versus 3 %, *p* = 0.644) rates were comparable in both patients’ cohorts. Postoperative therapy was applied in adjuvant intent to 42 (60 %) versus 51 (51 %) patients (*p* = 0.275). The 5-year RFS and CSS rates were 24 % versus 31 % (*p* = 0.169) and 42 % versus 55 % (*p* = 0.131), respectively.

**Conclusions:**

To our single-center experience, there is no significant effect of CRLM spreading (bilobar versus unilobar) on RFS and CSS rates. Bilobar CRLM are more likely to require extended multimodal efforts to achieve R0 resection.

**Electronic supplementary material:**

The online version of this article (doi:10.1007/s00384-012-1455-1) contains supplementary material, which is available to authorized users.

## Introduction

Surgical resection of colorectal liver metastases (CRLM) is the only treatment offering the prospect of long-term survival with reported 5-year survival rates of up to 58 % in highly specialized centers [1, 2]. Unfortunately, only 20–30 % of all patients with CRLM are resectable at the time of diagnosis even when assessed by an experienced hepatobiliary surgeon [3]. To increase the resectability rate, preoperative chemotherapy [4] and two-stage liver resections [5, 6] with portal vein occlusion (PVO) have been evaluated in the last decade within clinical trials and have proven benefit with an additional 25–75 % of selected patients achieving secondary resectability of their CRLM.

Bilobar distribution of CRLM is a major reason for primary unresectability and has been identified as negative prognostic parameter in previous studies [7, 8]. Patients with bilobar CRLM are significantly less often referred to a hepatobiliary surgeon although resection rates in referred patients did not differ between unilobar and bilobar disease [9]. However, with the implementation of interdisciplinary multimodal treatment strategies, the patient cohort suitable for liver resection nowadays consists not only of primarily resectable but also converted primarily irresectable patients. Accordingly, the extent of disease in patients deemed resectable quantified by Fong’s clinical risk score [10] increased during the last decade [11]. Furthermore, response to preoperative chemotherapy itself, which is mandatory in initially unresectable patients, is a positive predictor of survival [12]. In consequence, the current biological impact of bilobar hepatic spreading in patients with CRLM is unclear.

Therefore, the aim of the present single-center study was to evaluate the extent of oncological treatment applied for R0 resection in patients with bilobar versus unilobar CRLM and to compare the consecutive recurrence-free (RFS) and overall cancer-specific survival (CSS) after R0 resection considering interdisciplinary multimodal treatment options including intensified chemotherapy and extended liver resections.

## Patients and methods

### Study population

From January 2001 to December 2010, 170 patients underwent histopathologically confirmed complete (R0) resection (tumor-free resection margin ≥0.1 cm) of all CRLM at the Department of General and Visceral Surgery, University Medical Center Göttingen. One hundred thirteen of these patients presented with unilobar disease based on preoperative staging, but in 13 cases, additional CRLM in the contralateral liver lobe were detected at the time of liver resection. These 13 patients together with the 57 patients initially diagnosed with bilobar hepatic spreading (multiple bilobar CRLM or large involvement of both lobes by a central metastasis) represent the cohort of patients with bilobar CRLM and in this study were compared with the 100 patients with unilobar CRLM. Data on primary tumor treatment, CRLM-directed treatment strategies, perioperative outcome, and survival were prospectively collected in a database. The local ethics committee approved the study and all procedures were in accordance with the Helsinki Declaration of 1975, as revised in 1983.

Table 1 summarizes the clinicopathological baseline data concerning treatment of the primary colorectal cancer in all 170 patients. With median 59 (95 % CI 34–75) versus 63 (95 % CI 41–79) years, patients with bilobar CRLM were significantly younger than those with unilobar disease (*p* = 0.019). In one patient with locally advanced rectal cancer and synchronous bilobar CRLM, the neoadjuvant treatment did not comprise standard radiochemotherapy but intensified systemic chemotherapy with oxaliplatin alone according to the referring oncologist’s discretion. In six patients, histopathological workup identified tumor involvement at the resection margin in the primary rectal tumor specimen. However, staging prior to liver-directed therapy did not indicate local recurrence in any of these patients. Apart from age, there was no significant difference in relevant parameters between the two distinct study cohorts.

**Table 1:** Clinicopathological baseline data of primary tumor therapy

Parameter	Unilobar liver metastases *N* = 100	Bilobar liver metastases *N* = 70	*p* value
Age^a^	63 [41–79]	59 [34–75]	0.019
Gender			0.266
Female	44 (44 %)	24 (34 %)	
Male	56 (56 %)	46 (66 %)	
Primary tumor			0.755
Colon	56 (56 %)	41 (59 %)	
Rectum^b^	44 (44 %)	29 (41 %)	
Neoadjuvant therapy			0.116
Yes	18 (18 %)	6 (9 %)	
No	82 (82 %)	64 (91 %)	
T stage			0.535
<3	19 (19 %)	10 (14 %)	
≥3	81 (81 %)	60 (86 %)	
Nodal status			0.276
<1	52 (52 %)	30 (43 %)	
≥1	48 (48 %)	40 (57 %)	
Grading			0.245
<3	90 (90 %)	58 (83 %)	
≥3	10 (10 %)	12 (17 %)	
Resection status			0.083
0	99 (99 %)	65 (93 %)	
≥1	1 (1 %)	5 (7 %)	
UICC stage			0.082
≤2	33 (33 %)	17 (24 %)	
3	26 (26 %)	12 (17 %)	
4	41 (41 %)	41 (59 %)	
Adjuvant chemotherapy			0.136
Yes	37 (37 %)	18 (26 %)	
No	63 (63 %)	52 (74 %)	

^a^Expressed as median [95 % CI]

^b^Up to 16 cm from the anal verve measured by rigid rectoscopy

### Staging procedures and treatment algorithm for CRLM

Standardized pretherapeutical staging included clinical examination, chest-X-ray, abdominal computed tomography (CT) or magnetic resonance imaging (MRI), and serum level of carcinoembrionic antigen (CEA). Starting in January 2006, staging was routinely extended by thoracic CT and ^18^ F-fluorodeoxyglucose–positron emission tomography (FDG–PET). Prior to treatment onset in our department, patients were discussed in an interdisciplinary tumor board of medical as well as surgical oncologists, hepatobiliary surgeons, radiooncologists, and radiologists to define the individual multimodal treatment concept with special consideration of preoperative systemic chemotherapy and/or the need for two-stage hepatectomy. The latter was intended when the future remnant liver volume to body weight ratio was calculated to be less than 0.5 % [13].

Patients scheduled for preoperative systemic chemotherapy were re-evaluated every 3 months for secondary resectability. Tumor response to preoperative chemotherapy was measured by an experienced radiologist using the RECIST (Response Evaluation Criteria In Solid Tumors) criteria and classified as complete response, partial response, stable disease, or progressive disease [14]. Liver resection was performed according to established surgical standard operating procedures. Intraoperative ultrasound was used routinely prior to resection procedures to detect previously occult CRLM. In patients scheduled for two-stage hepatectomy, the first-stage procedure consisted of surgical exploration of the abdominal cavity, tumor clearance of the future remnant liver segments, and PVO on the side of predominant metastatic load. During the phase of PVO-induced liver hypertrophy, no additional chemotherapy was administered to avoid cumulative morbidity. The second-stage procedure with extended hemihepatectomy/trisectorectomy was planned 6–8 weeks after PVO. Postoperative liver insufficiency was defined by prothrombin time <50 % and serum bilirubin >50 μmol/l on post-op day 5 [15]. To estimate the risk of tumor recurrence, both the Fong score [10] and the Nordlinger score [16] were used. Furthermore, CRLM were classified according to the mTNM staging system as proposed by Gayowski et al. [8].

Until July 2008, the Association of Scientific Medical Societies in Germany (AWMF) interdisciplinary guidelines for the treatment of CRLM recommended a “wait and see” strategy after R0 resection of CRLM [17]. Despite this fact, we performed two phase II trials investigating the feasibility and safety of single or repeated anti-CEA radioimmunotherapy with ^131^I-labetuzumab (humanized anti-CEA immunoglobulin G1-subclass monoclonal antibody; Immunomedics Inc., Morris Planes, NJ, USA) after R0 resection of CRLM [18, 19]. Eligible patients with immunohistochemically proven high CEA expression on CRLM cells were enrolled while the study was open for recruitment. Other patients requesting postoperative therapy were treated by systemic chemotherapy according to the individual tumor board discretion and based on current treatment regimens as established for adjuvant treatment of primary colorectal cancer.

### Statistical analysis

Statistical analysis was performed using the Statistical Computing Software R [20]. Survival analysis was calculated from the date of liver resection on time to event data (i.e., time to cancer progression or time to cancer-specific death) using the R package survival. Survival data was visualized using Kaplan–Meier plots and significance was calculated using the Cox proportional hazards model. Significance for comparison between cohorts was calculated using Fisher’s exact test for categorical variables or variables that were discretized (e.g., gender, tumor stage, type of therapy) and using the Wilcoxon test for numeric variables (e.g., age, size of metastasis). *p* values <0.05 were considered significant.

## Results

### Preoperative treatment

Detailed data on CRLM treatment are displayed in Table 2. By preoperative staging, patients with bilobar CRLM had significantly more metastases than patients with unilobar hepatic spreading (*p* < 0.001). Furthermore, patients with bilobar CRLM were more often scheduled for preoperative 5FU-based systemic chemotherapy (*p* < 0.001) and had more often intensified cytostatic regimens combined with EGF and VEGF antibodies (*p* = 0.043). Additionally, the median number of applied chemotherapy cycles was higher in patients receiving EGFR/VEGF antibodies [eight cycles (95 % CI 3.2–24.4) versus four cycles (95 % CI 2–14.6); *p* < 0.001]. Because of CRLM progression, the initiated first-line regimen needed to be modified in equivalent proportions of both patients’ cohorts (11 % and 12 %, respectively; *p* = 1.0). Preoperative chemotherapy of CRLM resulted in clinical complete response, partial response, stable disease, and progressive disease in one (1 %), 28 (39 %), 32 (44 %), and 11 (15 %) patients, respectively. However, even the latter were deemed technically resectable according to restaging and scheduled for surgery based on excellent performance status and request.

**Table 2:** Data on treatment of colorectal liver metastases

Parameter	Unilobar liver metastases *N* = 100	Bilobar liver metastases *N* = 70	*p* value
No. of liver metastases preoperatively			<0.001
1	77 (77 %)	15 (21 %)^a^	
>1	23 (23 %)	55 (79 %)	
Median number of liver metastases pretherapeutical^b^	1 [1–4]	2 [1–7]	<0.001
Median diameter of largest liver metastasis pretherapeutical (cm)^b^	3 [1.1–9.5]	3 [1–10]	0.454
Preoperative chemotherapy			<0.001
No	74 (74 %)	24 (34 %)	
Yes	26 (26 %)	46 (66 %)	
5FU	6 (23 %)	3 (7 %)	
5FU + oxaliplatin	14 (54 %)	32 (70 %)	
5FU + irinotecan	6 (23 %)	11 (24 %)	
Antibody containing regimen			0.043
Yes	5 (19 %)	20 (43 %)	
No	21 (81 %)	26 (57 %)	
Number of cycles	5 [1.6–12.8]	6 [2–23]	0.675
More than one line			1.000
Yes	3 (12 %)	5 (11 %)	
No	23 (88 %)	41 (89 %)	
Type of resection^c^			0.061
Minor	54 (54 %)	27 (39 %)	
Major	46 (46 %)	43 (61 %)	
2-stage with PVO			
Yes	3	20	
No	97	50	
Morbidity rate	28 (28 %)	27 (39 %)	0.183
Cardiopulmonary	7	4	
Renal	1	0	0.009
Bilioma	4	10	┐
Liver insufficiency^d^	3	5	┘
Wound healing disorder	5	2	
Bleeding	2	0	
Reoperation	3	1	
Other	3	5	
Mortality rate	3 (3 %)	1 (1 %)	0.644
Number of liver metastases pathological			<0.001
1	73 (73 %)	17 (24 %)	
>1	27 (27 %)	53 (76 %)	
Median number of liver metastases pathological^b^	1 [1–6]	2 [1–6.3]	<0.001
Median diameter of largest liver metastasis pathological (cm)^b^	3.3 [0.9–11.3]	3.1 [0.7–10.7]	0.493
Resection margin			0.002
<1 cm	53 (53 %)	54 (77 %)	
≥1 cm	47 (47 %)	16 (23 %)	
mT stage [8]			<0.001
1	15 (15 %)	2 (2 %)	
2	65 (65 %)	6 (9 %)	
3	20 (20 %)	1 (1 %)	
4	0 (0 %)	61 (87 %)	
Fong score [10]			<0.001
0–2	48 (48 %)	60 (86 %)	
3–5	52 (52 %)	10 (14 %)	
Nordlinger score [16]			<0.001
0–2	42 (42 %)	10 (14 %)	
3–4	49 (49 %)	50 (71 %)	
5–6	9 (9 %)	10 (14 %)	
Adjuvant therapy			0.275
No	49 (49 %)	28 (40 %)	
Yes	51 (51 %)	42 (60 %)	
Systemic chemotherapy	25	11	
5FU	2 (8 %)	2 (18 %)	
5FU + oxaliplatin	13 (52 %)	6 (55 %)	
5FU + irinotecan	10 (40 %)	3 (27 %)	
Antibody containing regimen			0.645
Yes	3 (12 %)	2 (18 %)	
No	22 (88 %)	9 (82 %)	
Radioimmunotherapy	26	31	
Number of cycles			
Systemic chemotherapy	5 [2–12]	3 [1.3–11.8]	0.488
Radioimmunotherapy	2 [1–2]	1 [1–2]	0.205

^a^Six of these 15 patients had unilobar disease at initial staging

^b^Expressed as median [95 % CI]

^c^Minor: <hemihepatectomy; major: ≥hemihepatectomy

^d^Defined by prothrombin time <50 % and serum bilirubin >50 μmol/l on post-op day 5 [15]

### Surgical procedures

**Fig. 1:**
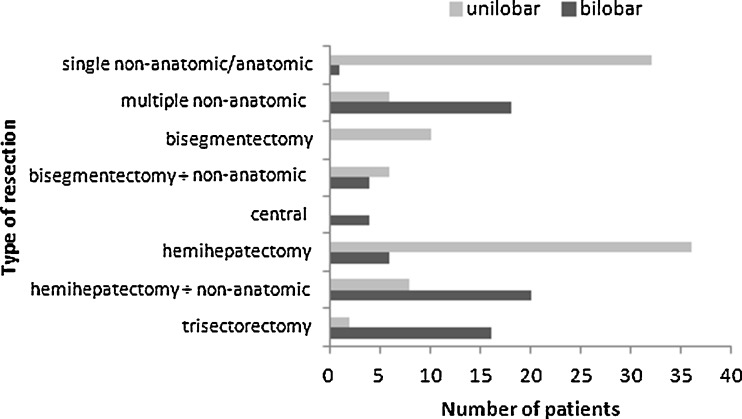
Type of resection in unilobar (*n* = 100) versus bilobar (*n* = 70) CRLM. The predominant surgical procedures were wedge resections and standard hemihepatectomies in patients with unilobar CRLM versus multiple wedge resections, extended hemihepatectomies, and trisegmentectomies in patients with bilobar CRLM. In nine patients with bilobar CRLM prior to preoperative chemotherapy, CRLM could only be detected in one liver lobe during surgery. These patients consecutively underwent unilobar resection

Differentiating between minor resections (<hemihepatectomy) versus major resections (≥hemihepatectomy), no significant difference in the extent of liver resections between the two study cohorts could be detected (*p* = 0.061). However, by analyzing the performed types of resection in detail (Fig. 1), this supposed equivalence could be traced back to a higher rate of standard hemihepatectomy in patients with unilobar CRLM (*n* = 36 versus *n* = 6; *p* < 0.001) while extended hemihepatectomies and trisectorectomies were performed significantly more often in patients with bilobar CRLM (*n* = 37 versus *n* = 10; *p* < 0.001). Furthermore, 29 % of patients with bilobar CRLM but only 3 % of those with unilobar disease (*p* < 0.001) had to be enrolled into two-stage procedures with PVO to ensure sufficient postoperative liver function. To note, in nine patients with bilobar CRLM prior to preoperative chemotherapy, CRLM could only be detected in one liver lobe during surgery. These patients consecutively underwent unilobar resection and had pathological mT1–3 stages according to Gayowski’s classification [8].

The overall morbidity rate was comparable in both study cohorts (28 % versus 39 %; *p* = 0.183) but bilioma formation and liver insufficiency were more frequent in patients with bilobar CRLM (*p* = 0.009). One patient with bilobar and three patients with unilobar CRLM died postoperatively because of liver failure after trisectorectomy (*n* = 2), acute hemorrhage (*n* = 1), and pneumonia (*n* = 1) resulting in a mortality rate of 2.4 %.

### Histopathological workup

Corresponding to the preoperative staging data, the histopathological workup including lamellation (1-cm slices) of the whole specimen identified more CRLM within the cohort of patients with bilobar hepatic spreading (*p* < 0.001). Furthermore, the proportion of patients with a minimal resection margin below 1 cm was significantly higher in the cohort with bilobar CRLM (*p* = 0.002). The use of clinical risk scores resulted in a heterogeneous pattern. According to the Nordlinger score [16], 15 % of patients with bilobar CRLM were classified as low risk while 85 % were deemed to have an intermediate/high risk of tumor recurrence. For patients with unilobar CRLM, nearly equivalent proportions of patients were grouped into low-risk (42 %) and intermediate/high-risk (58 %) categories. In contrast, the application of the Fong score [10] resulted in classification of 86 % of patients with bilobar CRLM into the low-risk group while for patients with unilobar CRLM, risk stratification results (48 % low and 52 % high risk) were comparable to those of the Nordlinger score.

### Postoperative treatment

The proportions of patients receiving postoperative adjuvant therapy (either radioimmunotherapy or systemic chemotherapy) were comparable (*p* = 0.275) between the two study cohorts. Similar to the preoperative setting, oxaliplatin was the predominant extension to 5FU-based chemotherapy.

### Survival

**Fig. 2:**
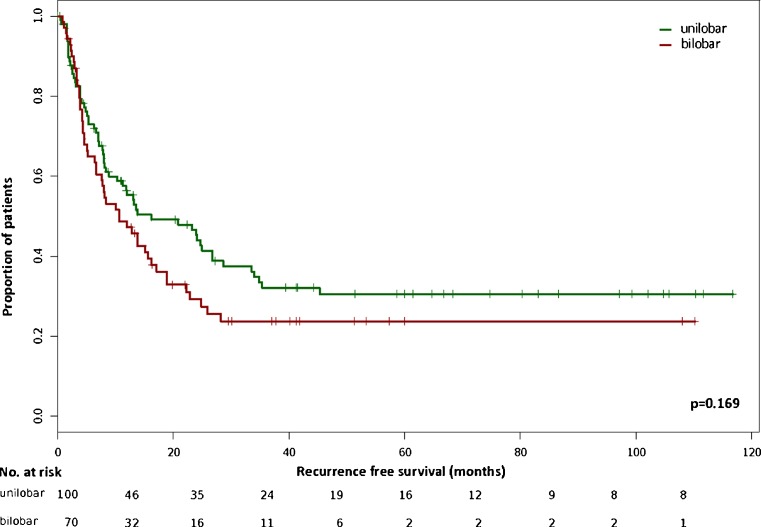
Kaplan–Meier curve for recurrence-free survival in patients following R0 resection of CRLM stratified by unilobar (*n* = 100) versus bilobar (*n* = 70) hepatic spreading

**Fig. 3:**
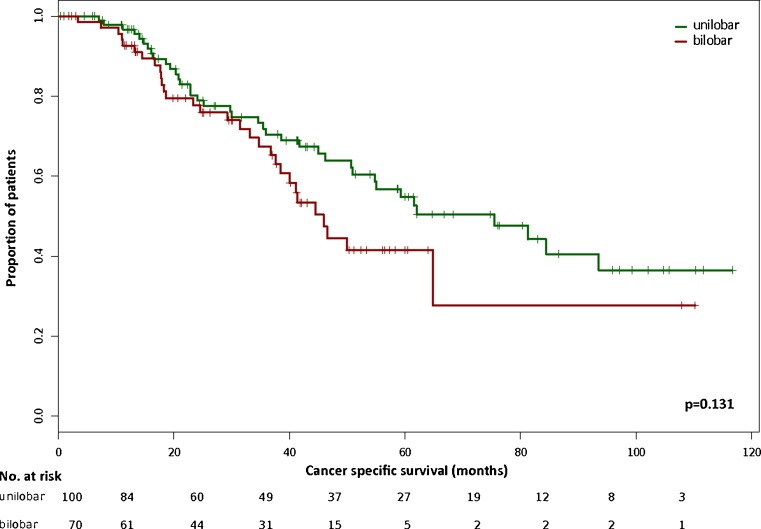
Kaplan–Meier curve of cancer-specific survival in patients following R0 resection of CRLM stratified by unilobar (*n* = 100) versus bilobar (*n* = 70) hepatic spreading

Median follow-up was 30.0 (95 % CI 3.3–76.7) months for patients with bilobar and 35.7 (95 % CI 1.6–108.2) months for patients with unilobar CRLM (*p* = 0.223). During follow-up, one patient died because of prostate cancer and eight patients died non-cancer related without evidence of recurrent disease. None of the patients developed metastatic recurrence more than 36 months after R0 resection of CRLM. Table 3 displays the localization of second metastatic recurrence for bi- versus unilobar CRLM. There was no significant difference in either recurrence rate (*p* = 0.327) or pattern of recurrence (*p* = 0.184). Median RFS was 10.6 (95 % CI 6.64–17.1) months for patients with bilobar and 16.1 (95 % CI 10.3–28.6) months for patients with unilobar CRLM. Consecutive median CSS was 45.9 (95 % CI 38.4–∞) months and 75.5 (95 % CI 50.9–∞) months, respectively. RFS rates (Fig. 2) in patients with bilobar CRLM (24 % at 3 years, 24 % at 5 years) were not significantly different from patients with unilobar CRLM (32 % at 3 years, 31 % at 5 years, *p* = 0.169). The consecutive CSS rates (Fig. 3) in patients with bilobar disease (67 % at 3 years, 42 % at 5 years) were also not significantly different to those in patients with unilobar CRLM (70 % at 3 years, 55 % at 5 years, *p* = 0.131). Because some patients had died for non-colorectal cancer reasons, we also calculated overall survival (OS) rates but could not observe a significant difference between bi- and unilobar CRLM either (*p* = 0.098). Univariate analysis for the study population (*n* = 170) revealed a trend in RFS (*p* = 0.06) and a significant difference in CSS (*p* = 0.007) for those patients with a FONG score ≤2 versus >2. In multivariate analysis with stratification for bilobar and unilobar CRLM neither for RFS nor for CSS, a significant difference could be detected (FONG*lobes ~ RFS HR = 0.7, *p* = 0.35; FONG*lobes ~ CSS HR = 0.9, *p* = 0.79). Furthermore, we performed a univariate analysis according to the stratification for synchronous versus metachronous CRLM. The analysis for the study population (*n* = 170) did not show significant differences for RFS (*p* = 0.179) and CSS (*p* = 0.972). The multivariate analysis with stratification for bilobar and unilobar CRLM revealed neither for RFS (synchronous/metachronous*lobes ~ RFS HR = 1.8; *p* = 0.13) nor for CSS (synchronous/metachronous*lobes ~ CSS HR = 1.2; *p* = 0.74) a difference.

**Table 3:** Data on second metastatic recurrence after R0 resection of colorectal liver metastases

Localization of second metastatic recurrence	Unilobar liver metastases *N* = 100	Bilobar liver metastases *N* = 70	*p* value
All	62 (100 %)	49 (100 %)	0.3273
Intrahepatic only	24 (39 %)	27 (55 %)	0.1842
Intra- and extrahepatic	6 (10 %)	5 (10 %)	
Extrahepatic only	32 (52 %)	17 (35 %)

Additionally, we performed a RFS and CSS analysis excluding the nine patients with solitary central CRLM involving both liver lobes. Again, there was no significant difference in RFS (*p* = 0.118) or CSS (*p* = 0.277) between both cohorts of bi- and unilobar CRLM.

## Discussion

Our single-center data in a consecutive series of R0-resected patients has not shown a relevant effect of type of CRLM spreading (bilobar versus unilobar) on RFS (24 % versus 31 %, *p* = 0.169) and CSS (42 % versus 55 %, *p* = 0.131). These results are encouraging especially for patients with bilobar CRLM formerly often deemed to have poor prognosis.

### Preoperative chemotherapy

We applied preoperative chemotherapy mainly for downsizing of initially unresectable CRLM but also to observe their chemoresponsiveness. However, chemotherapy was discontinued during liver hypertrophy following PVO as we speculated about increasing surgical morbidity and impaired liver volume gain. Although Chun et al. [21] demonstrated that the combination of preoperative chemotherapy and two-stage hepatectomy does not increase morbidity rates, the negative influence on liver hypertrophy is supported by delayed time to surgery in these patients [22, 23], and especially those patients with chemotherapy-induced steatosis experience significantly less hypertrophy of future remnant liver [23].

Patients with bilobar CRLM received significantly more often preoperative chemotherapy compared to patients with unilobar CRLM. This has also been shown by Kornprat et al. [24]. The median number of preoperative chemotherapy cycles for patients with bilobar CRLM in our study is in concordance with other authors [25, 26]. The use of EGFR and VEGF antibodies was limited to the late study period (2005–2010) when data on safety and efficacy emerged and German guidelines recommended more aggressive chemotherapy schedules to maximize the downsizing effect in initially unresectable CRLM [27]. Accordingly, chemotherapy combined with EGFR/VEGF antibodies was significantly more often applied to patients with bilobar CRLM.

### Surgical procedures

The predominant surgical procedures were wedge resections and standard hemihepatectomies in patients with unilobar CRLM. In contrast, multiple wedge resections, extended hemihepatectomies, and trisectorectomies were most often performed in patients with bilobar CRLM. Given that the majority of patients will experience recurrent metastatic disease which might be resectable in approximately 30 % of patients [28], we favored parenchymal-sparing wedge resections whenever possible. Gold et al. [29] have recently reported that this approach is associated with decreased mortality without negatively affecting survival.

Wicherts et al. [6] reported significantly poorer DFS and OS rates for patients treated within two-stage concepts including PVO compared to those with straightforward resection and without PVO. However, they included patients with positive resection margins into their analysis. In our own experience, RFS and CSS rates were comparable between straightforward resection and two-stage resection with PVO [30]. This was also shown by Mueller et al. [31]. Covey et al. [22] discussed a wide indication for two-stage resection including PVO to compensate the negative effects of preoperative chemotherapy. We would restrain such concept as PVO itself induces measurable intra- and extrahepatic tumor progression [32].

From the surgical aspect, multiple non-anatomic liver resections might be associated with a higher surgical risk than standard hemihepatectomies. As shown by our data (Fig. 1, Table 2), more complex procedures with a higher risk for intra-/postoperative complications were necessary in patients with bilobar CRLM.

### Survival

Recently, we have reported that patients with R0 resection of bilobar CRLM experience a significant survival benefit compared to R1/R2 resections followed by palliative chemotherapy [30]. These data have been confirmed by Brouquet et al. [25]. They further demonstrated that surgical exploration with R1/R2 resection followed by palliative chemotherapy is not inferior to palliative chemotherapy alone.

Now we focused on the impact of bilobar versus unilobar spreading of CRLM on RFS and CSS with respect to multimodal treatment options. We could demonstrate that neither RFS nor CSS nor OS were significantly different between patients with bilobar and unilobar CRLM. Within our cohort of bilobar CRLM, nine patients had solitary metastases with the majority being larger than 6 cm and substantial involvement of both liver lobes. However, as these might cause bias at least concerning RFS, we additionally performed RFS and CSS analyses excluding these nine patients. There was again no significant difference detectable in RFS and CSS between bilobar and unilobar CRLM. Kornprat et al. [24] had previously published their series of 98 patients with four or more CRLM. Fifty-five percent of these patients were treated by preoperative and 92 % by postoperative (adjuvant) chemotherapy. In univariate analysis, the type of CRLM spreading was not associated with survival. However, they included patients with positive resection margins and extrahepatic disease into their analysis. In contrast, Nikfarjam et al. [33] reported bilobar spreading of CRLM to be an independent negative prognostic parameter for long-term survival in the era of effective chemotherapy. Consistently to Kornprat et al. [24], they did not design a comparative study but performed uni- and multivariate analyses to identify prognostic parameters in their patient cohort of 64 patients. Furthermore, complete resection of all CRLM was performed only in 41 patients while in the remaining 23 patients radiofrequency ablation was used in addition to liver resection. Moreover, they also included patients with known extrahepatic disease (*n* = 5) and positive resection margins (*n* = 5) although Rees et al. [34] have demonstrated that positive resection margins are one of the strongest independent negative prognostic parameters. To the best of our knowledge, our data are the first focusing on the aspect of CRLM spreading in R0-resected patients in respect of multimodal treatment options.

Cummings et al. [35] have demonstrated that the benefit of liver resection reported by single-center experiences could be reproduced using a large population-based database. However, only 6.1 % of the identified 13,599 patients with CRLM underwent liver resection. Most likely a major reason of low resection rate is unawareness concerning the possibilities of modern multimodal treatment including advanced liver resection in patients with bilobar CRLM.

However, a non-critical use of both systemic chemotherapy and two-stage resections with PVL in patients with CRLM should be avoided. Although systemic chemotherapeutical options have evolved in the last decade in particular in terms of novel monoclonal EGFR and VEGF antibodies, a significant and substantial benefit of (intensified) systemic 5FU-based chemotherapy either pre- or postoperatively on survival has not been demonstrated for patients with resectable CRLM so far [36–38]. In contrast, advanced two-stage resections with PVL and intensified systemic 5FU-based preoperative chemotherapy in patients with primarily irresectable CRLM have shown to increase the secondary resection rate and thereby positively influence survival [4, 39].

## Conclusion

We demonstrated that the efforts and special risks of multimodal treatment in patients with bilobar CRLM are justified by encouraging survival rates when R0 resection becomes possible. Furthermore, there was no marked effect of CRLM spreading (bilobar versus unilobar) on RFS and CSS rates. Therefore, all patients with CRLM regardless of type of hepatic spreading need to be discussed within interdisciplinary tumor boards under participation of experienced hepatobiliary surgeons. All efforts should be undertaken to achieve R0 resection of all CRLM.

## Electronic supplementary material

Below is the link to the electronic supplementary material.

ESM 1 (PDF 21 kb)
