# Supplementary figures and images for: Bilobar spreading of colorectal liver metastases does not significantly affect survival after R0 resection in the era of interdisciplinary multimodal treatment
Source: Int J Colorectal Dis. 2012 Mar 21;27(10):1359–67. doi: 10.1007/s00384-012-1455-1 (PMC3449057; doi:10.1007/s00384-012-1455-1)

# OS lobes p=0.0979

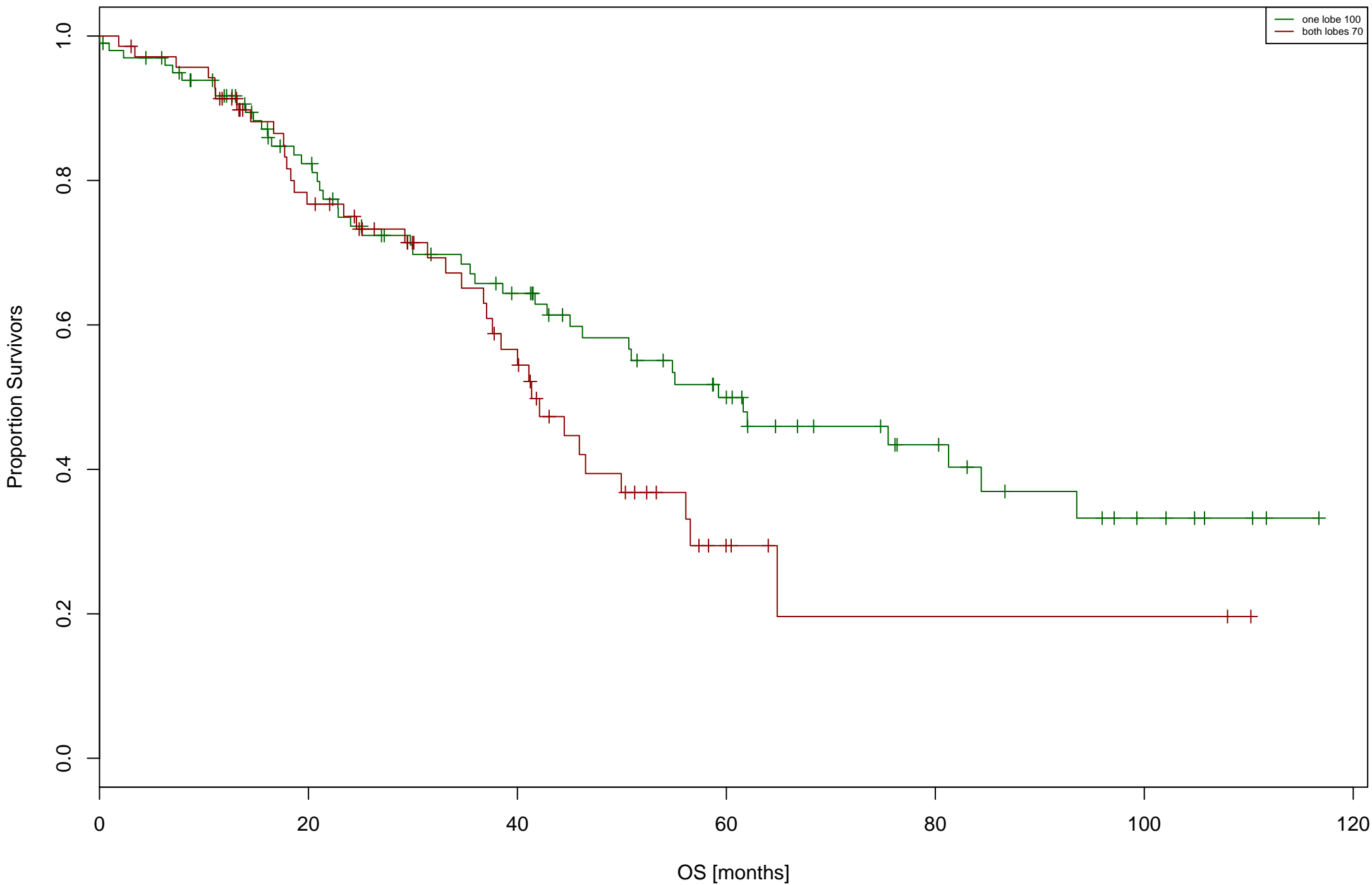

Supplement: Supplementary file 1 — (PDF 21 kb) [file 384_2012_1455_MOESM1_ESM.pdf]
